# Therapeutic efficacy and safety of JAK inhibitors in treating polymyositis/dermatomyositis: a single-arm systemic meta-analysis

**DOI:** 10.3389/fimmu.2024.1382728

**Published:** 2024-03-21

**Authors:** Chenhang Ma, Mengyao Liu, Yang Cheng, Xinchang Wang, Yu Zhao, Kailu Wang, Weijie Wang

**Affiliations:** ^1^ The Second Clinical Medical College of Zhejiang Chinese Medical University, Hangzhou, China; ^2^ The Second Affiliated Hospital of Zhejiang Chinese Medical University, Hangzhou, China

**Keywords:** Janus kinase inhibitors, idiopathic inflammatory myopathies, polymyositis, dermatomyositis, interstitial lung disease

## Abstract

**Introduction:**

We performed a single-arm meta-analysis to evaluate the efficacy and safety of JAK inhibitors in the treatment of dermatomyositis (DM)/ polymyositis (PM).

**Methods:**

Relevant studies from four databases were systematically searched until April 25, 2023. The primary endpoint was Cutaneous Dermatomyositis Disease Area and Severity Index (CDASI) and other outcomes were Manual Muscle Testing (MMT) and Creatine Kinase (CK). According to the type of JAK and medication regimen, we conducted subgroup analyses. The registration number in PROSPERO was CRD42023416493.

**Results:**

According to the selection criteria, we identified 7 publications with a total of 91 patients. Regarding skin lesions, the CDASI decreased by 17.67 (95% CI: -20.94 ~ -14.41). The CK increased by 8.64 U (95% CI: -28.25 ~ 45.53). About muscle lesions, MMT increased by 10.31 (95% CI: -2.83 ~ 23.46). Subgroup analysis revealed that different types of JAK inhibitors had various degrees of reduction. CDASI in patients treated with RUX had the lowest one [-20.00 (95% CI: -34.9 ~ -5.1)], followed by TOF [-18.29 (95% CI: -21.8 ~ -14.78)] and BAR [-11.2 (95% CI: -21.51 ~ -0.89)]. Additionally, the mean reduction in CDASI in patients treated with TOF alone was 16.16 (95% CI: -21.21 ~ -11.11), in combination with other immunosuppressants was 18.59 (95% CI: -22.74 ~ -14.45). For safety evaluation, one patient developed Orolabial HSV, and two patients developed thromboembolism events.

**Discussion:**

In summary, this meta-analysis demonstrated that JAK inhibitors can potentially treat DM/PM without severe adverse reactions.

**Systematic review registration:**

https://www.crd.york.ac.uk/PROSPERO/display_record.php?ID=CRD42023416493, identifier CRD42023416493.

## 1 Introduction

Idiopathic inflammatory myopathy (IIMs) affects the muscles, with symmetrical progressive myasthenia being the most prominent. DM, PM, and inclusion body myositis are the major subtypes of IIM. The main object of this study is DM/PM patients. DM is a type of inflammatory myopathy with no known cause. It often causes weakness in the muscles close to the body’s center. DM is also associated with other health issues outside of the muscles, such as skin problems like Gottron’s papules and heliotrope rash, as well as lung, digestive, joint, and heart problems (1, 2).

Systemic corticosteroids are the initial treatment for DM-related myopathy but have side effects. About half of patients taking glucocorticoid monotherapy relapse during tapering (3). Combining steroid-sparing immunosuppressive agents with glucocorticoid treatment can effectively lower the initial dose of glucocorticoids needed for remission induction, reduce the risk of relapse during glucocorticoid tapering, and minimize adverse effects of glucocorticoids. In cases of refractory IIM patients, a multi-target approach using both glucocorticoids and several steroid-sparing immunosuppressive agents has shown to be effective. However, there is complete control of disease in many patients with currently available therapies, as well as recurrence after achieving disease remission. Consequently, additional DM-modifying treatments must be developed urgently. Treatment with biologics, including rituximab and abatacept, is promising in some IIM patients. Recently, more and more case reports have been reported about the treatment of DM with JAK inhibitors. DM is driven by type I interferons. Genes regulated by interferon (IFN) are upregulated in DM. Specifically, type I and type II IFN-inducible genes are robustly expressed in myocytes of patients with DM, along with inflammation-related genes.

In view of the substantial evidence that IFN-regulated genes play a significant role in DM and Janus kinases (JAKs) play an obligate role in the signal transduction of IFNs, JAK inhibitors have been used in the treatment of DM. Both approved and being investigated, different types of JAK inhibitors exhibit distinct pharmacological activity on the four human JAK isoforms. Some of these inhibitors can potently inhibit JAK1 and/or TYK2, and several inhibit JAK1 and/or TYK2 potently and inhibit IFN types I and II accordingly (4).

Until now, there were no randomized clinical trials, systemic reviews, or meta-analyses on treating PM/DM by JAK inhibitors. Therefore, we conducted a single-arm meta-analysis to assess the effectiveness and safety of JAK in treating DM/PM.

## 2 Methods

### 2.1 Searching strategy and selection criteria

PRISMA checklist and Meta-Analysis guidelines were used to conduct the meta-analysis. From December 25, 2014 to April 25, 2023, we conducted a thorough search of four databases: PubMed, Embase, Web of Science, and the Cochrane Library. The relevant search terms are detailed in **Supplementary Appendix 1**.

The publications that were included in this meta-analysis needed to meet certain criteria: 1) Population: patients were diagnosed with DM/PM, including respective of the subtype and Anti-melanoma differentiation-associated protein (MDA) 5 (anti-MDA5)–positive amyopathic dermatomyositis (ADM)-associated interstitial lung disease (ILD) (ADM-ILD), 2) Intervention: patients were treated with JAK inhibitor or in combination with conventional as well as biologic immunosuppressant drugs, 3) Study type: retrospective studies, observational studies, letters (more than 10 patients), case series (more than 10 patients), 4) Primary outcomes: CDASI, MMT, 5) Secondary outcomes: CK, Forced vital capacity (FVC), Survival rate and adverse events (AEs).

**Figure 1:**
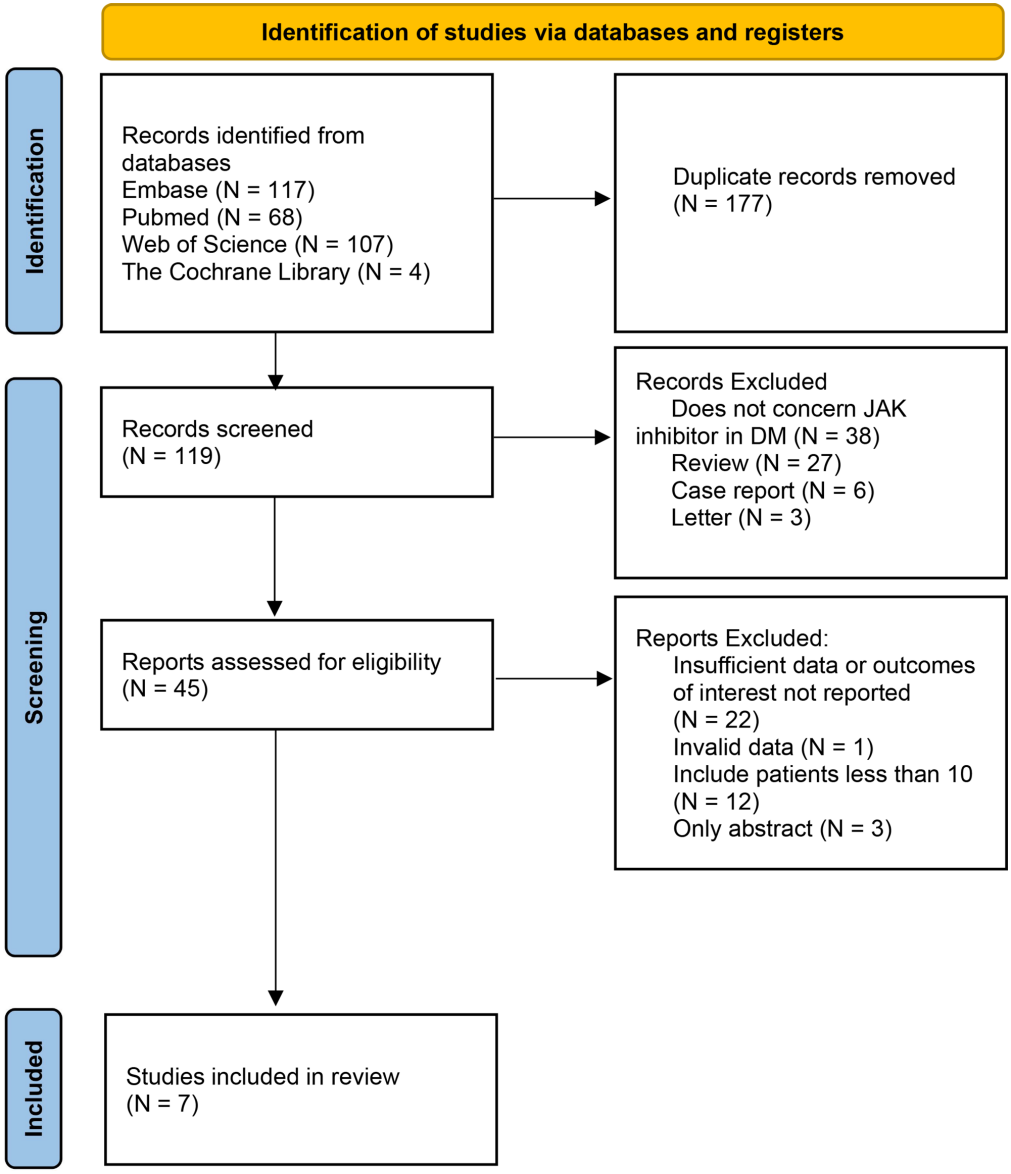
Flow diagram of meta-analysis for inclusion/exclusion of studies.

Conference abstracts without full text are excluded. Excluded publications lacked patient clinical characteristics, prior and/or concurrent therapies, or outcomes after JAK inhibitor treatment, as well as nonprimary case reports. If a patient appeared in multiple publications, they were only counted once (**Figure 1**).

### 2.2 Data extraction and quality assessment

The study was conducted independently by Chenhang Ma and Mengyao Liu to ensure consistency with similar analyses. In case of disagreement, Yang Cheng will join the discussion and resolve the disagreement.

Based on the included publications, the following information was extracted: the type of JAK inhibitors, number of patients, baseline demographics of patients, prior therapy, follow-up time, primary endpoints (CDASI, muscle enzyme (creatine kinase [CK], aldolase) levels, MMT). Results of the efficacy tests included the Cutaneous Dermatomyositis Disease Area and Severity Index (CDASI) activity score, manual muscle testing (MMT), muscle enzyme (creatine kinase [CK], aldolase) levels (a higher score shows more severe disease).

The data is extracted from text, tables, figures, or **Supplementary Materials**, and numeric values were obtained from chart images using WebPlotDigitizer.

### 2.3 Risk of bias assessment

The JBI quality assessment tool was used to evaluate the risk of bias in case series studies and retrospective studies. The quality of non-controlled trials was evaluated by the Newcastle-Ottawa Scale (NOS). We assessed each study and assigned a rating of low, high, or unclear risk of bias. To ensure consistency, we assessed all included publications. Any discrepancies were resolved through consensus.

### 2.4 Statistical analysis

We used the Polling random model (I-V heterogeneity) to calculate effect estimates, and the statistical method was no Standard statistic. The dichotomous outcomes were summarized by using Risk Ratio based on the 2x2 contingency tables from two studies (5, 6). The risk ratio was calculated by the ratio of deaths to survivors, the continuous outcomes as standardized mean differences with 95% credible intervals. To assess the heterogeneity, we used the chi-squared test and *I^2^* statistic. A *P*-value of less than 0.05 suggested a statistical difference. Furthermore, we conducted a sensitivity analysis to assess the combined results. Lastly, we evaluated the possible publication bias by performing beggs and eggers tests. In the subgroup analysis, CDASI data were grouped according to different drug types, drug regimens, and median follow-up months. Forest maps were made by STATA 16.0 (StataCorp LLC, College Station, TX 77845) for analysis and comparison.

## 3 Results

### 3.1 Study selection and characteristics

The initial database search yielded 296 relevant published studies. After deletion of duplicate literature and preliminary browsing the abstracts, 45 studies were retained. The next step involved a thorough evaluation of the full-text articles that were left after the initial screening process, 38 studies were excluded because of insufficient data or outcomes of interest not reported, unavailable full text, small sample size (less than 10 patients), or non-chemotherapy drugs.

Finally, 7 studies with 91 patients met the inclusion criteria and were included in this meta-analysis (3–11). The flowchart of the selection process is shown in **Figure 1**. The details of each included study are described in **Table 1**.

**Table 1:** Characteristics of the studies included in the meta-analysis.

Study, Year	Type	Mean age,years (mean ± SD)	Median follow-up,months	Intervention	Prior therapy	Endpoints	Total no. patients(lesions)
Fan 2022 (6)	Retrospective observation study	55.42 ± 2.2	6	Tofacitinib	CS	FVC, CK, Survival rate, Ferritin level	26
Chen 2019 (5)	Prospective single-center open-label study	47.6 ± 13.8	6	Tofacitinib	Methylprednisolone	FVC, CK, Survival rate, Ferritin level	18
Landon-Cardinal 2023 (7)	Open label pilot, single center study	55.4	3	Ruxolitinib (n = 4); Baricitinib (n = 12)	CS, MTX, HCQ, IVIG, RTX, AZA	CDASI, MMT-8, CK, IFN-a	16
Min 2022 (11)	Retrospective observation study	44.9	27	Tofacitinib	HCQ, prednisone, MTX, mycophenolate, IVIG, lenalidomide, isotretinoin, dapsone	CDASI	11
Paik 2021 (10)	Prospective open label pilot study	45.6	3	Tofacitinib	MTX, AZA, MMF, tacrolimus, HCQ, RTX, cyclophosphamide, IVIG	CDASI, MMT, CK, IFN, CXCL9, CXCL10, TIS	10
Paik 2022 (9)	Prospective open label pilot study	45.6	5	Tofacitinib	MTX, AZA, MMF, tacrolimus, HCQ, RTX, cyclophosphamide, IVIG	CDASI, MMT, CK, IFN, CXCL9, CXCL10, TIS	10
Le Voyer 2021 (4)	Retrospective monocentric study	9.1	6	Ruxolitinib (n = 7); Baricitinib (n = 3)	CS, MTX, IVIG	CMAS, MMT	10

CS, corticosteroids; FVC, Forced vital capacity; CK, Creatine Kinase; MTX, methotrexate; HQC, hydroxychloroquine; IVIG, intravenous immunoglobulin; RTX, rituximab; AZA, azathioprin; CDASI, Cutaneous Dermatomyositis Disease Area and Severity Index; MMT-8, Manual Muscle Testing-8; IFN, interferon; MMF, Mycophenolate Mofetil; CXCL9, Chemokine (C-X-C motif) ligand 9; TIS, total improvement score; CMAS, Childhood Myositis Assessment Scale.

### 3.2 Quality assessment

Three non-randomized studies was assessed using the Newcastle–Ottawa Scale (NOS), which categorized studies into three dimensions based on eight items, including population selection, comparability, and outcome (cohort studies) or exposure (case–control studies) evaluation (4). Other studies were assessed by the JBI Critical Appraisal Checklist for Case Series. Details are in **Tables 2**, **3**.

**Table 2:** Quality assessment of the studies included in the meta-analysis (JBI Critical Appraisal Checklist for Case Series for included retrospective studies).

Study, Year	Q1	Q2	Q3	Q4	Q5	Q6	Q7	Q8	Q9	Q10	Total
Landon-Cardinal 2023	2	2	2	2	2	2	2	2	0	2	18
Min 2022	2	2	2	2	2	2	2	2	2	2	20
Paik 2021	2	2	2	2	2	2	2	2	2	2	20
Paik 2022	2	2	2	2	2	2	2	2	2	2	20

Numbers Q1-Q10 in heading signified: Q1, were there clear criteria for inclusion in the case series? Q2, was the condition measured in a standard, reliable way for all participants included in the case series? Q3, were valid methods used for identification of the condition for all participants included in the case series? Q4, did the case series have consecutive inclusion of participants? Q5, did the case series have complete inclusion of participants? Q6, was there clear reporting of the demographics of the participants in the study? Q7, was there clear reporting of clinical information of the participants? Q8, were the outcomes or follow-up results of cases clearly reported? Q9, was there clear reporting of the presenting site(s)/clinic(s) demographic information? Q10, was statistical analysis appropriate?.

**Table 3:** Quality assessment of the studies included in the meta-analysis (Newcastle–Ottawa Scale (NOS) for included non-randomized studies).

Study, Year	I	II	III	IV	V	VI	VII	VIII	Total
Le Voyer 2021	1	1	1	0	0	1	1	1	6
Fan 2022	1	1	1	0	1	1	1	1	7
Chen 2019	1	1	1	0	1	1	1	0	6

Numbers I-VIII in heading signified: I, representatives of the exposed cohort; II, selection of the non-exposed cohort; III, ascertainment of exposure; IV, demonstration that outcome of interest was present at the start of the study; V, comparability of cohorts on the basis of the design or analysis; VI, assessment of the outcome; VII, was follow-up long enough for outcomes to occur? VIII, adequacy of follow-up of cohorts.

### 3.3 Skin lesion

**Figure 2:**
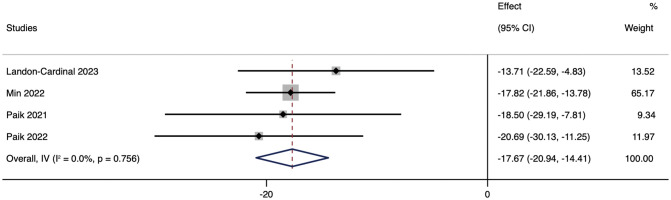
Forest plot about the pooled results of CDASI. The kind of effect size is a standardized mean difference.

As the primary outcome, most of the literature included in this review used CDASI as an essential indicator of JAK inhibitors improving the degree of skin lesions. These studies showed that CDASI decreased after the treatment of DM with JAK inhibitors. Heterogeneity was low (*p* = 0.756; *I²* = 0%), using the random effects model. Analysis showed that the CDASI score decreased by 17.67 (95% CI: -20.94 ~ -14.41) after treatment.(**Figure 2**).

**Figure 3:**
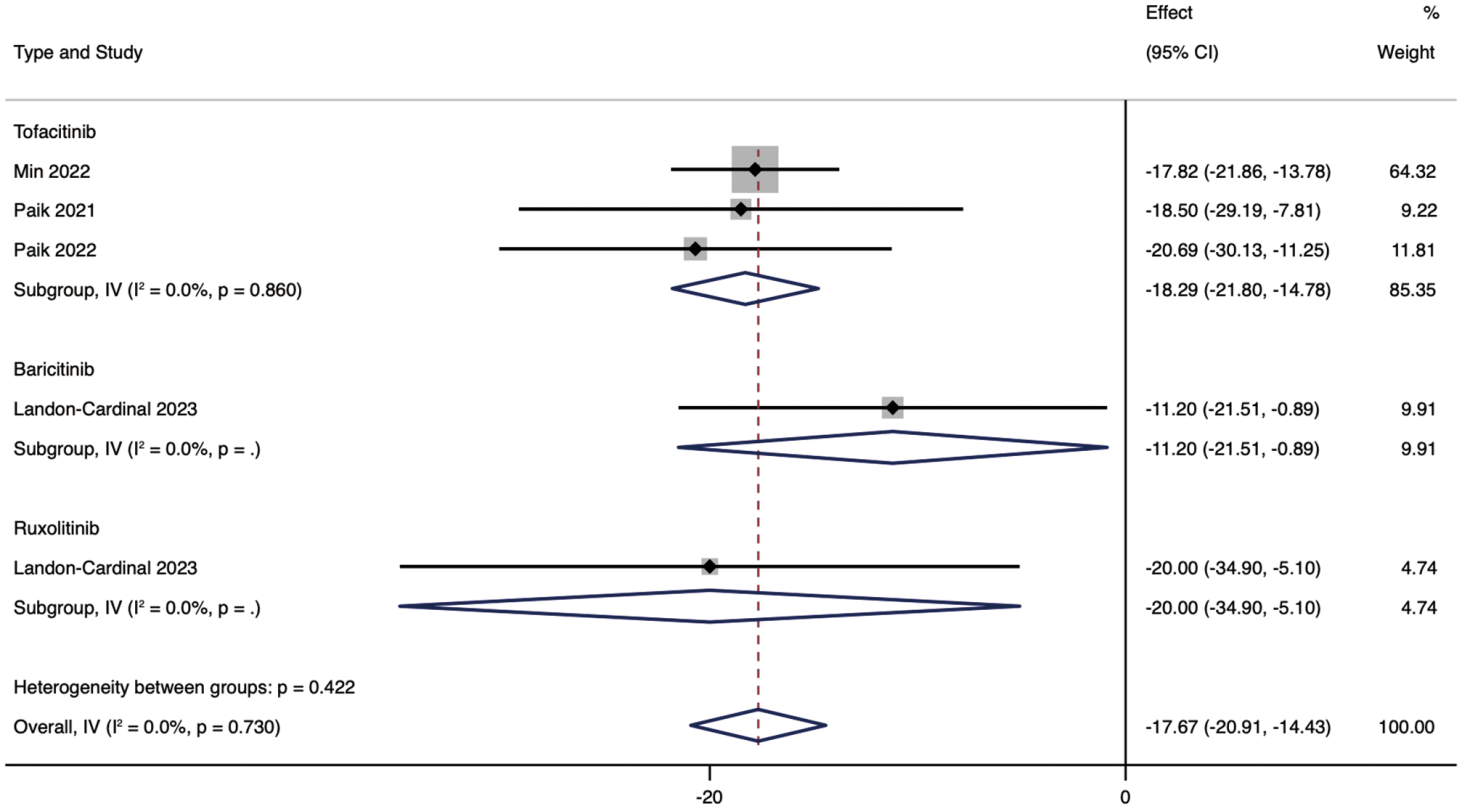
Forest plot showing changes in CDASI by drug type. The kind of effect size is a standardized mean difference.

We performed subgroup analyses based on drug types and medication regimens to further analyze JAK inhibitors’ efficacy. Subgroup analysis showed that, according to different drug types of JAK inhibitors, the mean reduction in CDASI in patients treated with RUX was 20.00 (95% CI: -34.9 ~ 5.1). The mean decrease in CDASI in patients treated with RUX was 20.00 (95% CI: -34.9 ~ -5.1). The efficacy of TOF was not different from that of RUX, and the CDASI score was improved by 18.29 (95% CI: -21.8 ~ -14.78). The efficacy of BAR was inferior to that of RUX and TOF, with a mean reduction in CDASI of 11.2 (95% CI: -21.51 ~ -0.89) in patients in the BAR group (**Figure 3**).

**Figure 4:**
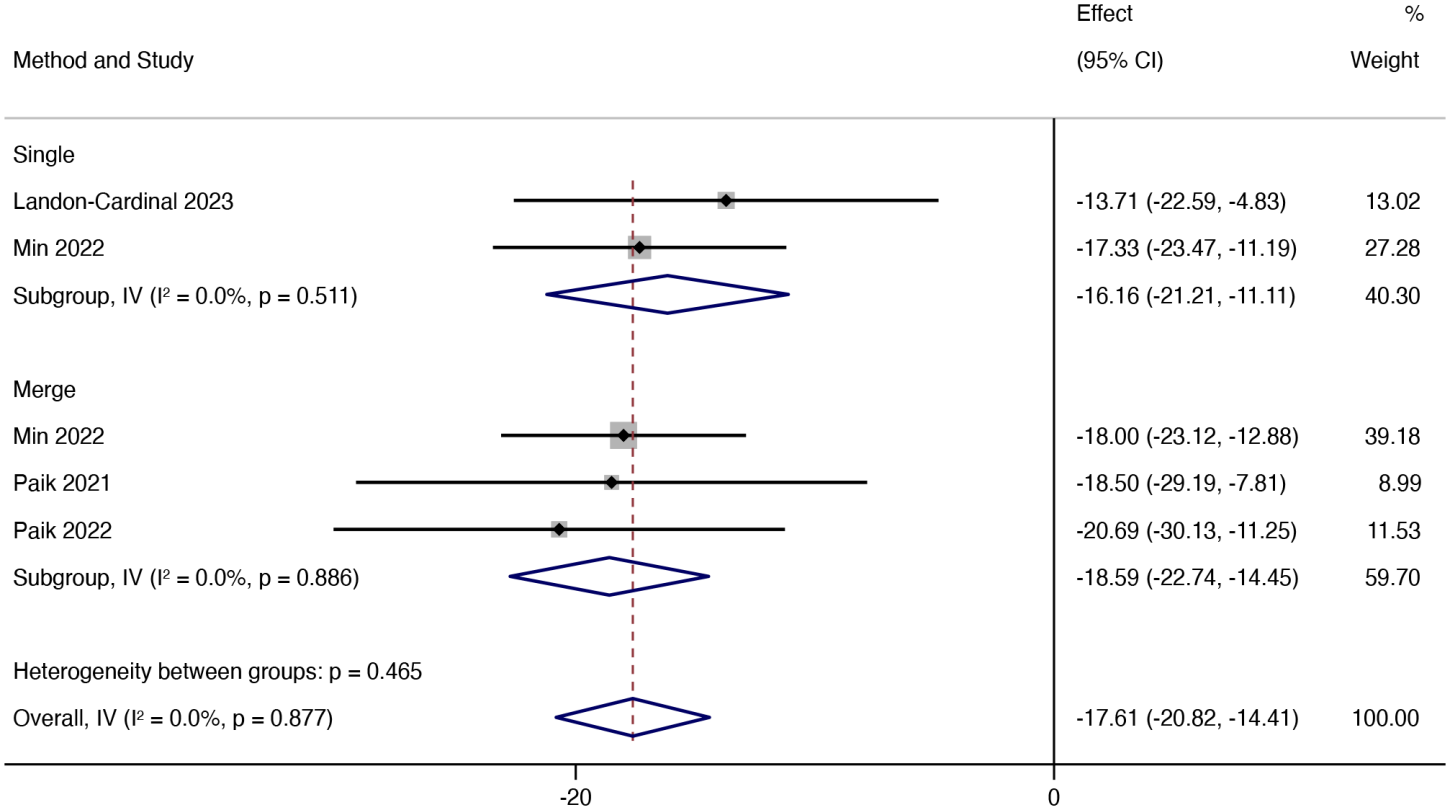
Forest plot showing changes in CDASI by medication regimen. The kind of effect size is a standardized mean difference.

Then, further analysis was conducted based on whether or not JAK inhibitors were combined. The results showed that combining other immunosuppressants was more effective than JAK inhibitors alone, but the difference in efficacy was slight. The mean reduction in CDASI in patients treated with TOF alone was 16.16 (95% CI: -21.21 ~ -11.11), the mean decrease in CDASI in combination with other immunosuppressants was 18.59 (95% CI: -22.74 ~ -14.45) (**Figure 4**).

**Figure 5:**
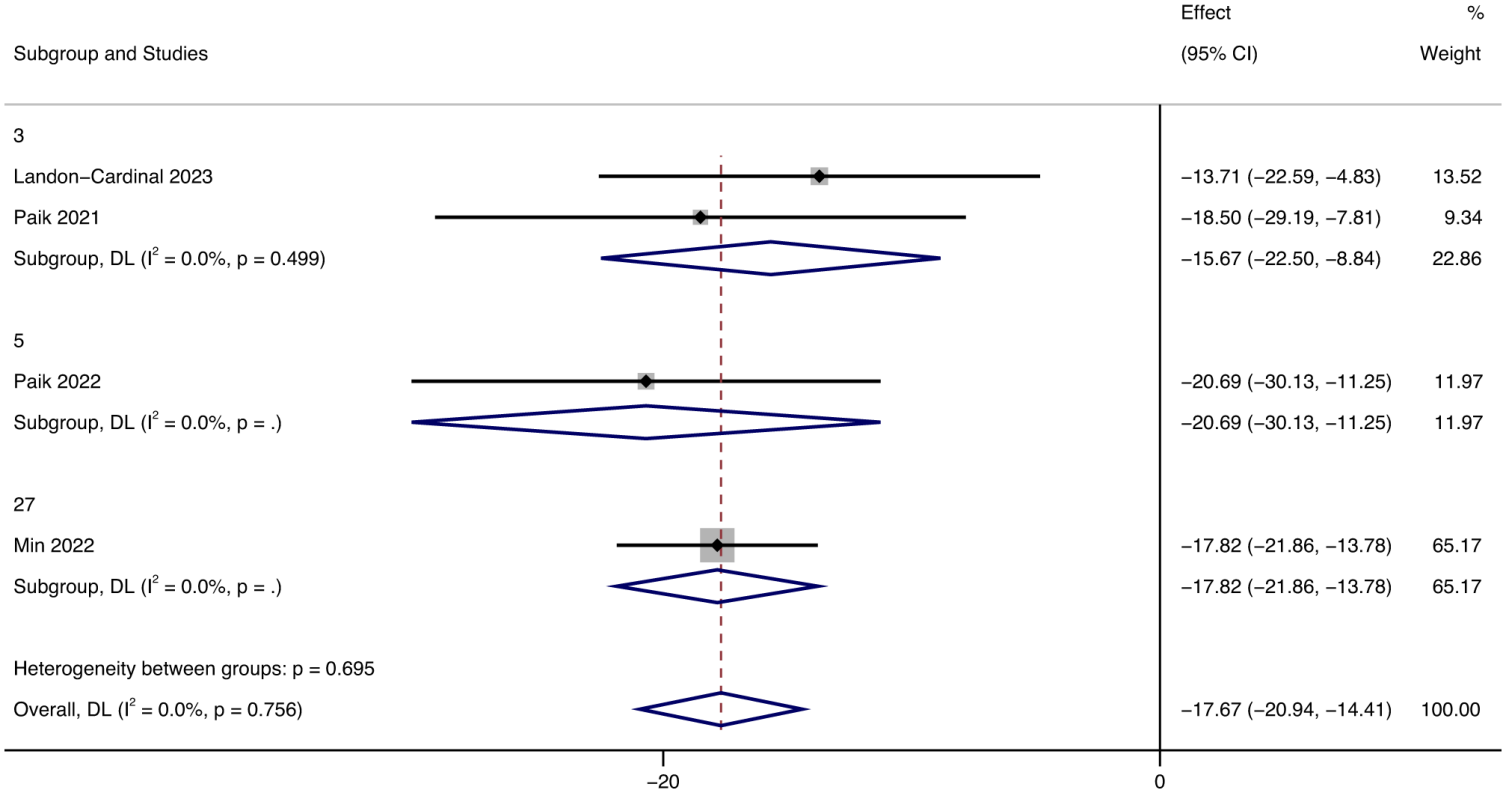
Forest plot showing changes in CDASI by Median follow-up months. The kind of effect size is a standardized mean difference.

Additionally, we discussed the influence of different follow-up times on CDASI and the results showed that in different follow-up times (3, 5, 27 months respectively), the improvement of the patient’s condition was more obvious with the increase of follow-up time (**Figure 5**).

### 3.4 Muscle condition

**Figure 6:**
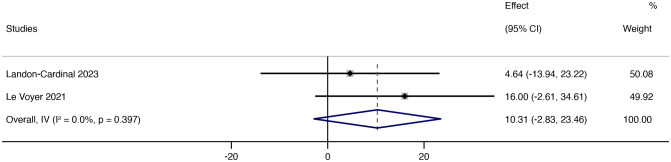
Forest plot about the results of MMT. The kind of effect size is a standardized mean difference.

After treatment with JAK inhibitors, MMT increased by 10.31 (95% CI: -2.83 ~ 23.46). The heterogeneity was low (*p* = 0.397; *I²* = 0%) using the random effects model (**Figure 6**). From the forest plot results of MMT, JAK inhibitor therapy can potentially improve muscle strength in patients.

### 3.5 CK

Since the units related to CK in the included literature were UI and U, we conducted a subgroup analysis of the results of CK, and the grouping was based on the units of the results. Despite being in different units, the results all showed decreased CK. In terms of UI, after treatment with JAK inhibitors, CK levels decreased by 174.5 UI (95% CI: -580.20 ~ 231.20) on average. In terms of U, after treatment with JAK inhibitors, CK levels increased by 8.64 U (95% CI: -28.25 ~ 45.53, *p* = 0.1, *I²* = 63%) on average.

**Figure 7:**
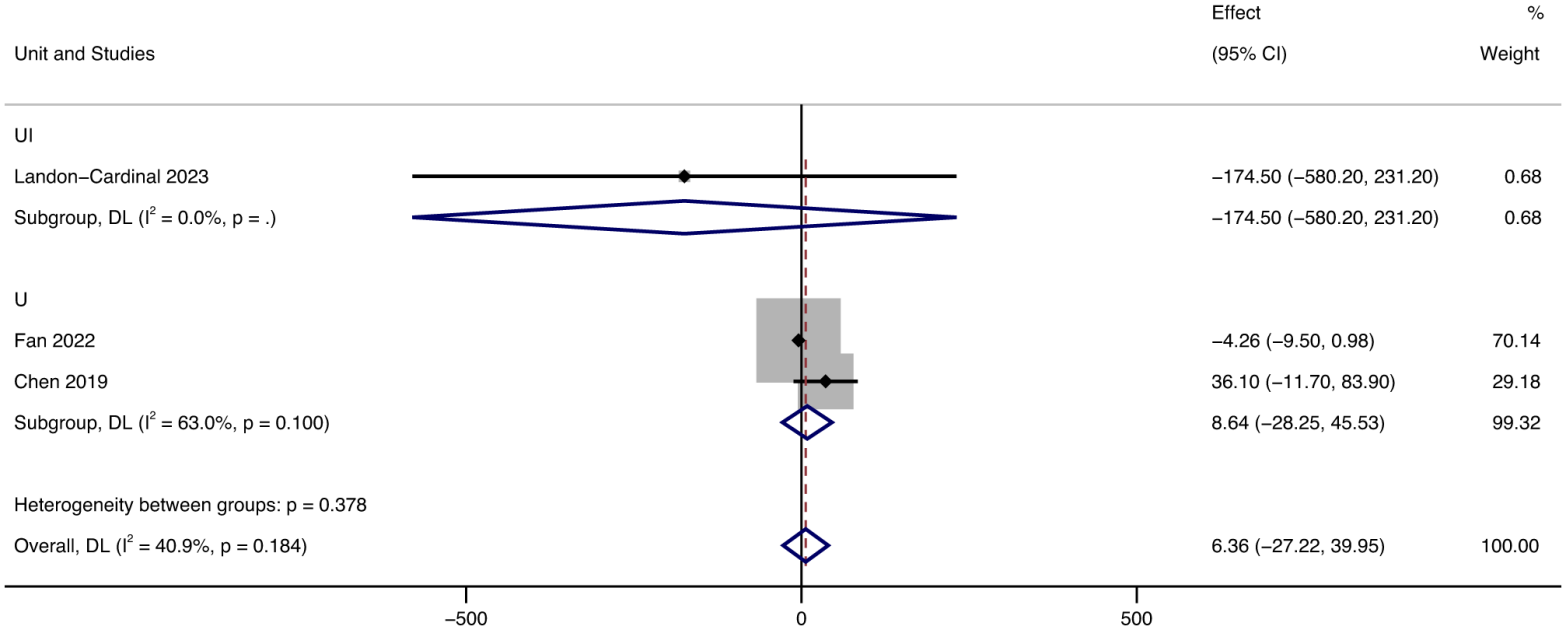
Forest plot about the results of CK. The kind of effect size is a standardized mean difference.

In the literature, with CK as the index, there were two units of U and UI. Since only one article in the literature has UI as the unit, we display the results with U as the main unit. Analyzing the forest map of CK results, we found that the heterogeneity of outcomes in U is high (*I²* = 63%), which we believe is caused by the small number of literatures in U (**Figure 7**).

### 3.6 ADM-ILD

According to the relevant studies, anti-melanoma differentiation-associated gene 5 (MDA-5) antibodies are associated with DM. Additionally, it is associated with the development of interstitial lung disease (ILD). (Anti-MDA5) - positive amyopathic dermatomyositis (ADM) - associated interstitial lung disease (ILD) (hereafter, ADMILD) is a rapidly progressive and life-threatening disease. The current therapies used to treat this disease do not improve the prognosis of patients apparently.

Type I interferon gene expression is JAK - STAT pathway stimulated, thus, IFN1 is relevant to Anti-MDA-5 antibody-positive dermatomyositis (12). The JAK inhibitor has been used recently to treat refractory dermatomyositis. There has been some progress treating recalcitrant cutaneous lesions and ILD (5).

**Figure 8:**
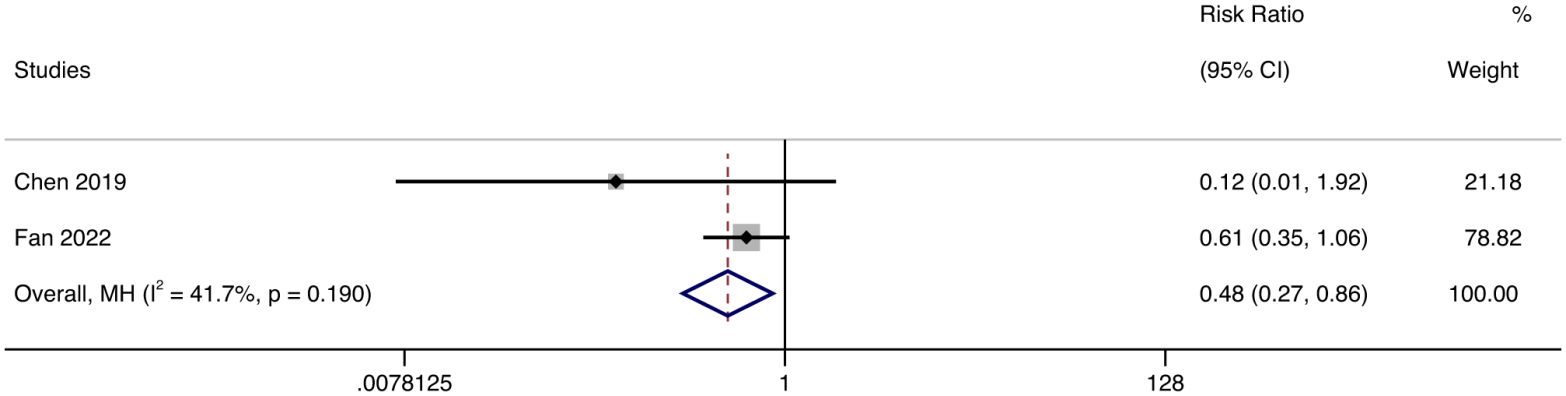
Forest plot about the results of survival rate.

We included all eligible ADM-ILD patients, analyzed their survival rate and FVC index data, made forest maps using STATA16.0 software, and analyzed whether JAK inhibitors were effective in these patients. Due to the different types of literature included and the differences in treatment options other than JAK inhibitors, we used a random effect model to minimize the impact of heterogeneity on the results. Random effect model meta-analysis showed that the survival rate of the TOF group was higher than that of the control group (*RR* = 0.48, 95% CL: 0.27 ~ 0.86, *p* = 0.19) (**Figure 8**). Of all the patients included in the publications, only one patient has been reported to have died due to anti-melanoma differentiation associated protein 51 with progressive interstitial lung disease (7). Based on the survival of patients reported in all publications, it is tentatively believed that JAK inhibitors have a slight chance of death after DM treatment, which has great potential to improve the survival rate of patients. And we don’t think JAK inhibitors have serious life-threatening side effects.

**Figure 9:**
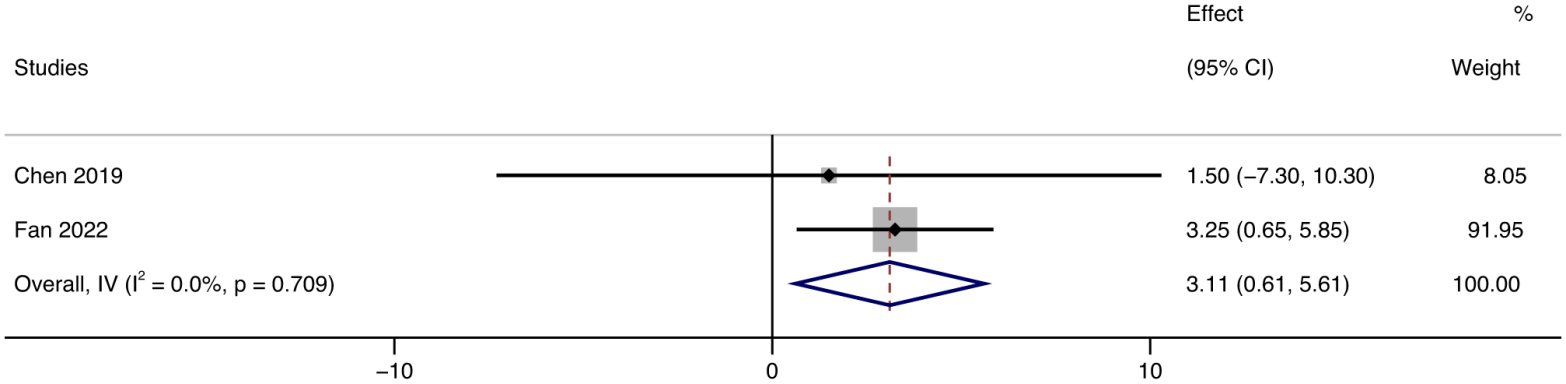
Forest plot about the results of FVC. The kind of effect size is a standardized mean difference.

FVC (percent of predicted value) in the tofacitinib group was also considerably improved over time, increasing by 3.11% (95% CI: 0.61 ~ 5.61, *p* = 0.709, *I²* = 0%) (**Figure 9**).

### 3.7 Adverse impact

Since the number of adverse events reported in the included publication was insufficient for statistical analysis, we adopted a tabular approach to counting adverse events during JAK inhibitor therapy (**Table 4**). The side effects of the treatment may include elevated levels of creatinine, liver transaminases, and lipids. In addition, there may be an initial decrease in the number of lymphocytes, neutrophils, natural killer cells, and platelets, which could increase the risk of thromboembolism events and infectious diseases, including tuberculosis and viral infections such as herpes zoster (1). In addition, adverse events occurred more frequently in patients with AMD-ILD compared to other patients (3).

**Table 4:** Adverse events of the studies included in the meta-analysis.

Study	AE	Number of deaths	Remarks
Fan 2022 (6)	5 patients were clinically considered to have pulmonary fungal infection.	12	Only adverse events in the TOF group are reported here.
Chen 2019 (5)	1 patient had urinary tract infection (Escherichia coli); 1 patient had liver function abnormality; 1 patient had possible invasive fungal infection.	0	Only adverse events in the TOF group are reported here.
Landon-Cardinal 2023 (7)	One patient developed thromboembolic events and another, treated concomitantly with mycophenolate mofetil, presented with febrile neutropenia both requiring transient hospitalization in the intensive care unit. No patients developed cancer.	1	One patient died (anti-melanoma differentiation- associated protein51 with progressive interstitial lung disease), and 2 patients relapsed (muscle disease).
Min 2022 (11)	Only one patient developed Orolabial HSV (herpes simplex virus).	0	
Paik 2021 (10)	No adverse events occurred.	0	
Paik 2022 (9)	No serious adverse events occurred and no subject discontinued tofacitinib.	0	
Le Voyer 2021 (4)	No adverse events occurred.	0	

### 3.8 Sensitivity analysis

To assess the impact of each individual study on the overall results, a sensitivity analysis was conducted by removing one study at a time. The analysis showed that none of the individual studies had a significant influence on the pooled results with 95% confidence intervals. Therefore, the results of this meta-analysis were found to be relatively reliable overall (**Supplementary Figure 1**).

### 3.9 Publication bias

To detect publication bias and assess the validity of the results, eggers and beggs tests were employed. According to the tests, all the results did not show publication bias of homicide (**Supplementary Figures 2–4**).

Possible publication bias includes dizziness, headache, gastrointestinal reactions (nausea, diarrhea), nasopharyngitis, and infections (especially respiratory and urinary tract infections).

## 4 Discussion

JAK inhibitors have the potential to treat DM/PM and have a specific reference value in the treatment of refractory DM/PM because JAK inhibitors have a disease-modifying effect in rheumatic diseases. It was not found that the patients documented in the literature included in this systematic literature review improved with further first-line and second-line treatments (such as methotrexate, mycophenolate mofetil, azathioprine, etc.), including corticosteroids and other immunomodulatory agents (such as IVIG). The assessment of DM outcomes is multifaceted. We assessed the therapeutic effects of JAK inhibitors in DM by observing skin lesions, muscle strength, and some serological indicators (e.g., CDASI, MMT, CK). This is the first single-arm study to evaluate the efficacy and safety of JAK inhibitors in the treatment of polymyositis/dermatomyositis. We included not only typical DM/PM patients but also those diagnosed with ADM-ILD, and it was shown that JAK inhibitors could improve the condition of these patients to a certain extent.

We emphasize that patients with significant skin symptoms demonstrated the most remarkable clinical improvements. CDASI, as the primary outcome, can accurately reflect the progress of the patient’s skin lesions. After using JAK inhibitors, patients’ CDASI scores decreased by an average of 17.67. To further understand the effect of JAK inhibitors, we conducted subgroup analyses according to the different drug types and drug regimens. According to the type of drug, we divided the patients into three groups who had been treated with tofacitinib (JAK1/2/3 inhibitor), baricitinib (JAK1/2 inhibitor), and ruxolitinib (JAK1/2 inhibitor) during their illness. Then, the improvement of CDASI scores in each group was compared by subgroup analysis. The results showed that the efficacy of RUX was the best; the average CDASI score was reduced by 20. The effectiveness of TOF was not significantly different from that of RUX; the average CDASI score was decreased by 18.29, while the effect of RUX was relatively not noticeable. We believe that the various therapeutic effects of the three groups of drugs have a lot to do with the mechanism of the drugs. Tofacitinib inhibits JAK1/JAK3 and, to a lesser extent, JAK2/TYK2. Ruxolitinib interferes JAK1 and JAK2, with moderate activity against TYK2. Baricitinib inhibits JAK1 and JAK2 (1). BAR inhibits the fewest pathways, which may be the reason for its relatively poor efficacy. In theory, TOF inhibition has a broader range, and the effectiveness should be the best. However, the results of the subgroup analysis showed that the efficacy of TOF and RUX was similar, and even RUX was better. We believe this is because so few RCT articles on the treatment of DM/PM with JAK inhibitors have been published, resulting in an insufficient number of patients included, and the results may have specific errors. In addition, compared with other drugs, TOF was usually chosen for the treatment of DM in more studies. The researchers were more inclined to choose, and the number of patients with other drugs was smaller than in the TOF group, leading to some margin of error in the results. Thus, it would be persuasive to conduct further clinical trials to compare the effectivity of TOF and RUX. According to the different treatment regimens, the patients were divided into two groups: JAK inhibitor monotherapy and combined with other immunosuppressive drugs. The results show that the therapeutic effect of combination therapy is better than that of JAK inhibitor alone, so we suggest that other drugs can be used in combination therapy when JAK inhibitor therapy is used. After using the JAK inhibitor, muscle strength increased in most patients, and MMT scores increased by 10.31 points on average. In terms of CK, different units showed different results, after using JAK inhibitors, the CK level of patients measured in UI decreased by 174.5 UI, while the CK level of patients measured in U increased by 8.64U. Due to the small number of patients with CK as the endpoint, so whether JAK inhibitors could improve CK levels in patients is questionable. However, there is no doubt that JAK inhibitors could improve MMT in patients, which indicated that JAK inhibitors were effective in improving the muscle condition of patients. To further evaluate the efficacy of Jak inhibitors, we enrolled patients with AMD-ILD. We found that patients treated with JAK inhibitors had a higher survival rate and FVC improvement than conventional treatment. The survival rate of the TOF group was higher than that of the control group (*RR* = 0.48, 95%CL: 0.27 ~ 0.86, *p* = 0.19). FVC (percentage of predicted value) in the tofacitinib group was also improved by 3.11 (95%CL: 0.61 ~ 6.61, *p* = 0.709).

JAKs are intracellular tyrosine kinases that activate STATs and regulate cytokine-mediated immune responses. Dysregulated JAK-STAT signaling is implicated in autoimmune diseases, inflammation, allergic reactions, and cancer (2). JAK plays its role through a variety of pathways, through which it can regulate cell proliferation, activation, and other activities. According to the available research, there are a total of 4 Jaks and 7 STATS. The abnormal functioning of JAK-STAT pathways is linked to several immune diseases. This further supports the possibility and potential of JAK inhibitors in treating DM. Only a few JAK inhibitors are approved for DM. Baricitinib (inhibit JAK1/2), upadacitinib (inhibit JAK1), ruxolitinib (inhibit JAK1/2), and tofacitinib (inhibit JAK1/2/3) are the approved JAK inhibitors for autoimmune diseases (1).

### 4.1 Limitations and considerations for the future

This study is a single-arm systemic meta-analysis of JAK inhibitor therapy and DM/PM, which is relatively comprehensive and considers many factors. However, there are still some limitations. As there are few RCTs of JAK inhibitors in DM/PM, most of which are still experimental and the results have not yet been published, related RCTS are still in progress, and there is no complete experimental data to publish related articles. Therefore, retrospective studies and other types of studies with relatively high levels of evidence were included in this paper. It was changed from a meta-analysis to a single-arm study, and the data extraction and comparison were performed as far as possible to maintain uniform standards after screening. Because there are few RCTs in treating DM/PM with JAK inhibitors, and most related RCTs are still in the experimental stage, there is no complete experimental data to publish related articles. Therefore, retrospective studies and other research types with relatively high evidence levels are included in this paper. The study was changed from meta-analysis to single-arm systemic meta-analysis, and the data extraction and comparison were carried out with uniform standards as far as possible after screening. We tried to keep this error to a minimum and conducted a careful literature quality assessment of all included studies. Due to the small number of literature that could be included, the number of patients included has also become one of the limitations of this article. The number of patients is only 91, so the data and conclusions may be somewhat biased. However, we have carried out careful quality evaluation, sensitivity analysis, heterogeneity evaluation, and publication bias evaluation in this paper to make the data and conclusions as reliable as possible. The results of our single-arm study suggest that JAK inhibitors are effective in treating DM/PM and can improve patient prognosis. The use of JAK inhibitors can improve the survival rate and prognosis of patients diagnosed with ADM-ILD. In addition, drug-induced side effects are rarely reported.

In the analysis of outcomes for anti-MDA5 patients, there were some differences in the treatment regimen of the patients included in the two studies (5, 6). All patients were treated with TOF, but some of them were exposed to other immunosuppressants (e.g., Cyclosporine, Mycophenolate mofetil, Cyclophosphamide, Azathioprine, etc.). Moreover, in one of the studies, the control group was treated with tacrolimus (6). Meanwhile, the doses of glucocorticoid were also different. Therefore, there may be heterogeneity in the results. To reduce heterogeneity, a random effects model was used for analysis.

Nevertheless, due to a small amount of data, confirmation of this conclusion requires more RCTs. Moreover, we believe there is a need to ensure more uniform and clear clinical indicators to evaluate the condition of DM/PM patients.

## 5 Conclusion

Our meta-analysis demonstrates the efficacy and safety of JAK inhibitors in patients with DM/PM, providing evidence for its future clinical application. Nevertheless, due to a small amount of data, confirmation of this conclusion requires more RCTs.

## Data availability statement

The original contributions presented in the study are included in the article/**Supplementary Material**. Further inquiries can be directed to the corresponding author.

## Author contributions

CM: Writing – review & editing, Writing – original draft, Software, Methodology, Formal analysis, Data curation. ML: Writing – review & editing, Writing – original draft, Supervision. YC: Writing – review & editing, Data curation. XW: Writing – review & editing, Supervision. YZ: Writing – review & editing, Software, Data curation. KW: Writing – review & editing, Data curation. WW: Writing – review & editing, Supervision, Conceptualization.
