# Supplementary material for: Therapeutic efficacy and safety of JAK inhibitors in treating polymyositis/dermatomyositis: a single-arm systemic meta-analysis
Source: Front Immunol. 2024 Mar 21;15:1382728. doi: 10.3389/fimmu.2024.1382728 (PMC10991784; doi:10.3389/fimmu.2024.1382728)
Supplement: Supplementary file 1 [file DataSheet_1.pdf]

frontiers

Supplementary Material

1 Supplementary Appendix 1

Full search strategy (April 25, 2023): PubMed

Searches
#1 "Polymyositis"[MeSH Terms] OR ("Polymyositides"[Title/Abstract] OR "myositis multiple"[Title/Abstract] OR "multiple myositis"[Title/Abstract] OR (("Myositis"[MeSH Terms] OR "Myositis"[All Fields] OR "myositides"[All Fields]) AND "Multiple"[Title/Abstract]) OR "polymyositis idiopathic"[Title/Abstract] OR (("Idiopathic"[All Fields] OR "idiopathically"[All Fields] OR "idiopathics"[All Fields]) AND "Polymyositides"[Title/Abstract]) OR "idiopathic polymyositis"[Title/Abstract] OR (("Polymyositis"[MeSH Terms] OR "Polymyositis"[All Fields] OR "Polymyositides"[All Fields]) AND "Idiopathic"[Title/Abstract]) OR "polymyositis ossificans"[Title/Abstract] OR "ossificans polymyositis"[Title/Abstract]) OR ("Dermatomyositis"[MeSH Terms] OR ("Dermatopolymyositis"[Title/Abstract] OR "Polymyositis-Dermatomyositis"[Title/Abstract] OR "Polymyositis-Dermatomyositis"[Title/Abstract] OR (("Dermatomyositis"[MeSH Terms] OR "Dermatomyositis"[All Fields]) AND "adult type"[Title/Abstract]) OR "adult type dermatomyositis"[Title/Abstract] OR "dermatomyositis childhood type"[Title/Abstract] OR "childhood type dermatomyositis"[Title/Abstract] OR "juvenile dermatomyositis"[Title/Abstract] OR "dermatomyositis juvenile"[Title/Abstract] OR "juvenile myositis"[Title/Abstract] OR "myositis juvenile"[Title/Abstract])) <br>#2 "baricitinib"[Supplementary Concept] OR ("3-azetidineacetonitrile"[All Fields] OR "Olumiant"[Title/Abstract] OR (("baricitinib"[Supplementary Concept] OR "baricitinib"[All Fields]) AND "phosphate"[Title/Abstract]) OR (("baricitinib"[Supplementary Concept] OR "baricitinib"[All Fields]) AND "phosphate salt"[Title/Abstract]) OR ("3-azetidineacetonitrile"[All Fields] AND "1"[All Fields] AND "ethylsulfonyl"[Title/Abstract] AND "3"[All Fields] AND ("4"[All Fields] AND ("7H-pyrrolo"[All Fields] AND "2 3 d"[All Fields] AND "pyrimidin-4-yl"[All Fields]) AND "1h pyrazol 1 yl"[All Fields]) AND ("phosphated"[All Fields] OR "phosphates"[MeSH Terms] OR "phosphates"[All Fields] OR "phosphate"[All Fields] OR "phosphatic"[All Fields] OR "phosphating"[All Fields] OR "phosphation"[All Fields] OR "phosphatized"[All Fields]) AND "1 1"[All Fields]) OR "INCB028050"[Title/Abstract] OR "INCB-028050"[Title/Abstract] OR "LY3009104"[Title/Abstract] OR "LY-3009104"[Title/Abstract]) OR "delgocitinib"[Supplementary Concept] OR ("tofacitinib"[Supplementary Concept] OR ("tasocitinib"[Title/Abstract] OR "tofacitinib citrate"[Title/Abstract] OR "Xeljanz"[Title/Abstract] OR "cp 690 550"[Title/Abstract] OR "CP690550"[Title/Abstract] OR "CP-690550"[Title/Abstract] OR "CP-690550"[Title/Abstract] OR "cp 690 550"[Title/Abstract])) OR ("ruxolitinib"[Supplementary Concept] OR (("3R"[All Fields] AND "3 cyclopentyl 3"[All Fields] AND ("4"[All Fields] AND ("7H-pyrrolo"[All Fields] AND "2 3 d"[All Fields] AND "pyrimidin-4-yl"[All Fields]) AND "pyrazol-1-yl"[All Fields]) AND "propanenitrile"[Title/Abstract]) OR "ruxolitinib phosphate"[Title/Abstract] OR

(("ruxolitinib"[Supplementary Concept] OR "ruxolitinib"[All Fields]) AND "monophosphate"[Title/Abstract]) OR (("ruxolitinib"[Supplementary Concept] OR "ruxolitinib"[All Fields] OR "INCB-18424"[All Fields]) AND "phosphate"[Title/Abstract]) OR "Jakavi"[Title/Abstract] OR "incb018424 phosphate"[Title/Abstract] OR (("ruxolitinib"[Supplementary Concept] OR "ruxolitinib"[All Fields] OR "INCB-018424"[All Fields]) AND "salt"[Title/Abstract]) OR "Jakafi"[Title/Abstract] OR (("ruxolitinib"[Supplementary Concept] OR "ruxolitinib"[All Fields]) AND "as phosphate"[Title/Abstract]) OR (("ruxolitinib"[Supplementary Concept] OR "ruxolitinib"[All Fields] OR "INCB-018424"[All Fields]) AND "phosphate"[Title/Abstract]) OR "opzelura"[Title/Abstract] OR "INCB-018424"[Title/Abstract] OR "INC-424"[Title/Abstract] OR "INCB-18424"[Title/Abstract] OR "INC424"[Title/Abstract] OR "INCB018424"[Title/Abstract])) OR ("Janus Kinase Inhibitors"[MeSH Terms] OR ("inhibitors janus kinase"[Title/Abstract] OR "kinase inhibitors janus"[Title/Abstract] OR "jak inhibitors"[Title/Abstract] OR "inhibitors jak"[Title/Abstract] OR "janus kinase inhibitor"[Title/Abstract] OR "inhibitor janus kinase"[Title/Abstract] OR (("kinase s"[All Fields] OR "phosphotransferases"[MeSH Terms] OR "phosphotransferases"[All Fields] OR "Kinase"[All Fields] OR "kinases"[All Fields]) AND "inhibitor janus"[Title/Abstract]) OR "jak inhibitor"[Title/Abstract] OR "inhibitor jak"[Title/Abstract]))

#3 ("randomized controlled trial"[Publication Type] OR "controlled clinical trial"[Publication Type] OR "randomized"[Title/Abstract] OR "randomised"[Title/Abstract] OR "placebo"[Title/Abstract] OR "drug therapy"[MeSH Subheading] OR "randomly"[Title/Abstract] OR "trial"[Title/Abstract] OR "groups"[Title/Abstract]) NOT ("animals"[MeSH Terms] NOT "humans"[MeSH Terms])

#4 #1 AND #2 AND #3

#5 "animals"[MeSH Terms] NOT "humans"[MeSH Terms]

#6 #4 NOT #5

2 Supplementary Figures

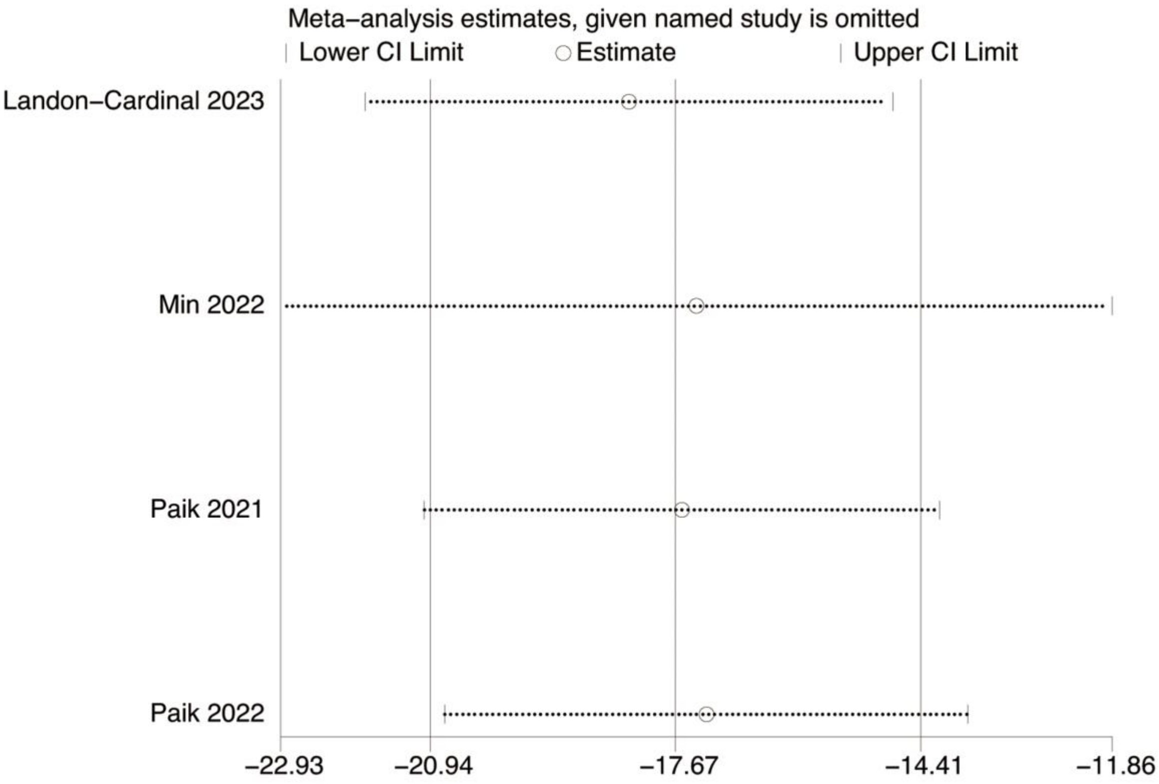

Supplementary Figure 1. Sensitive analysis of the effects of JAK inhibitors on CDASI.

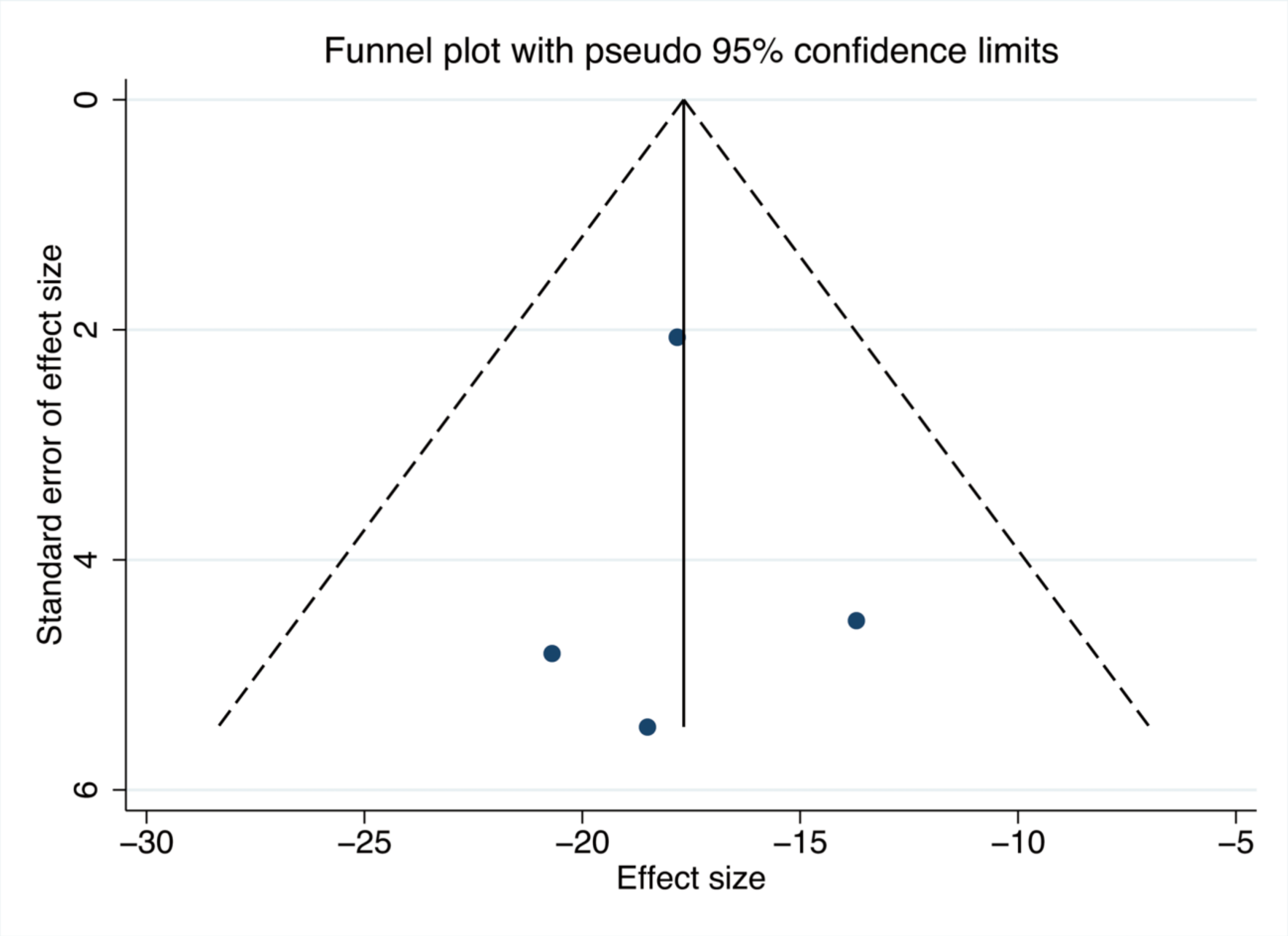

Supplementary Figure 2. The funnel plots of the effects of JAK inhibitors on CDASI.

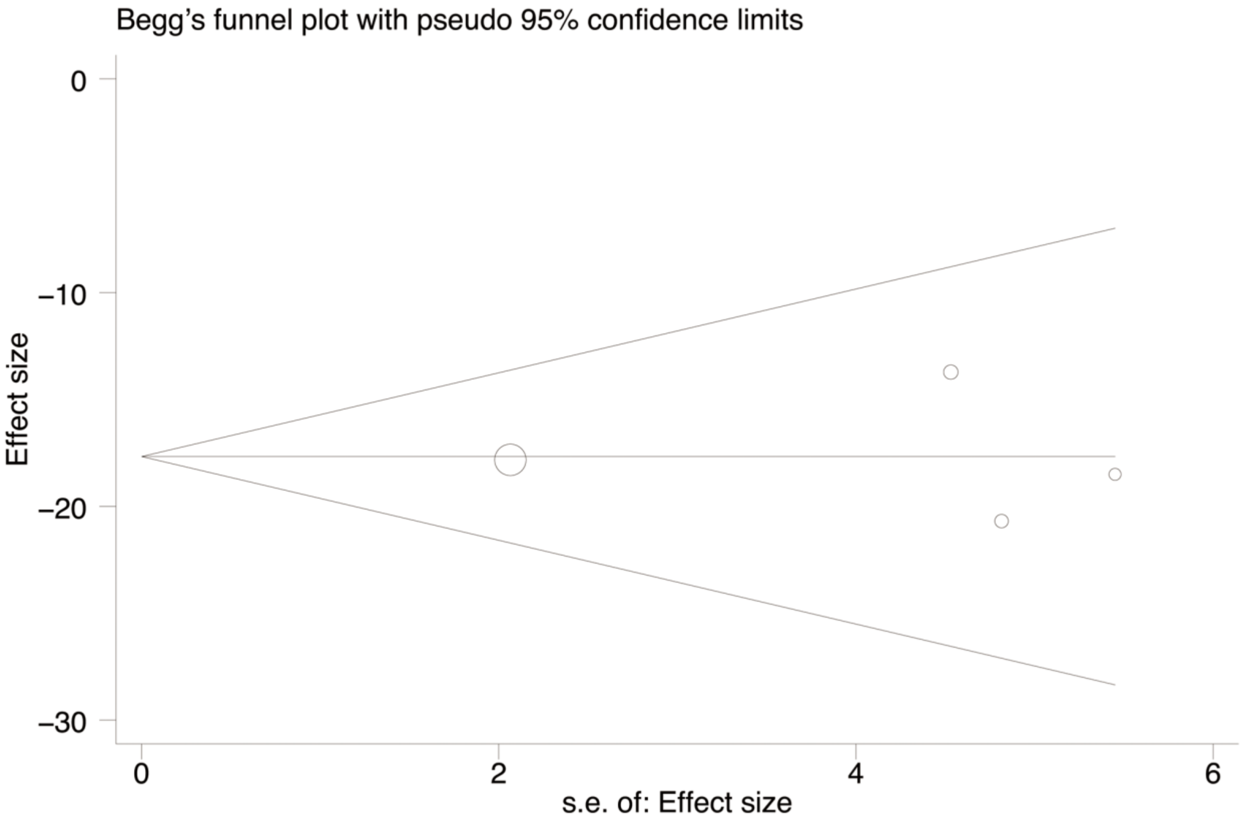

Supplementary Figure 3. The Begg's test of the effects of JAK inhibitors on CDASI.

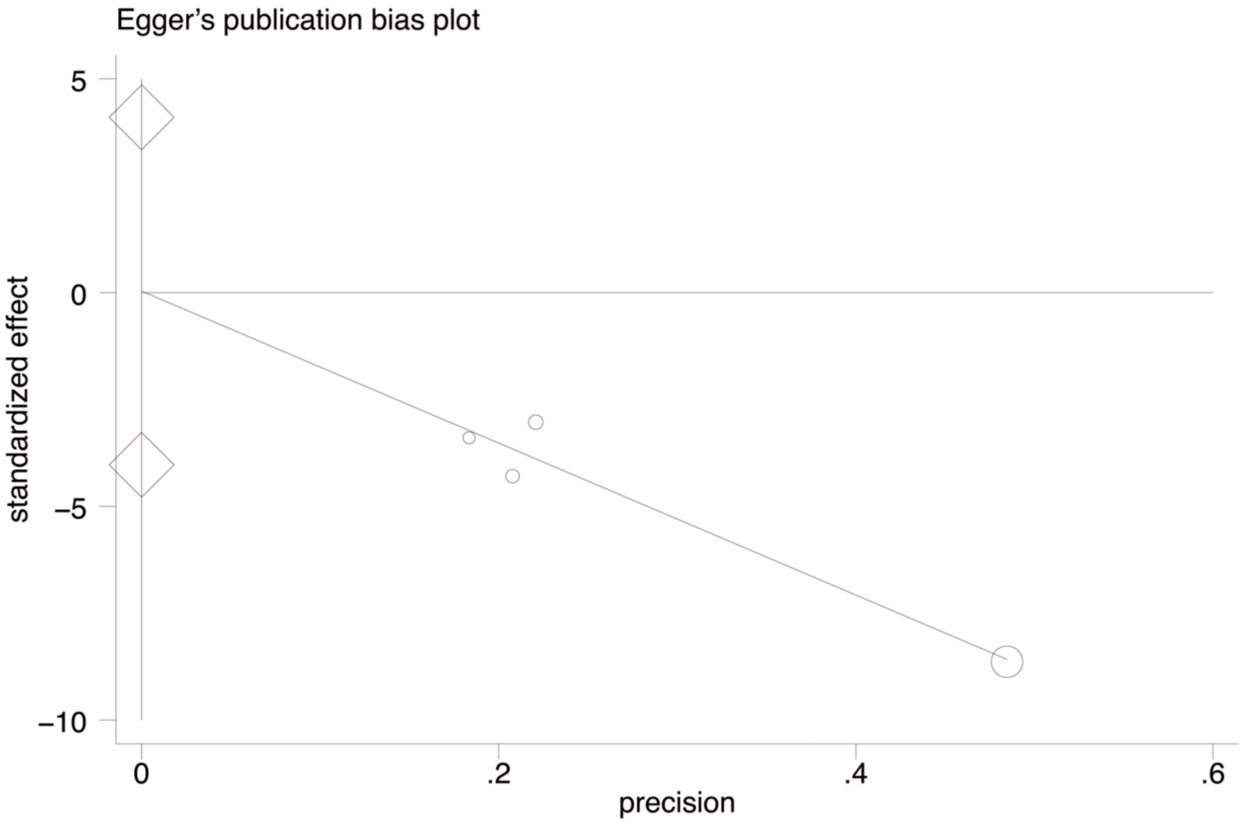

Supplementary Figure 4. The Egger's test of the effects of JAK inhibitors on CDASI.

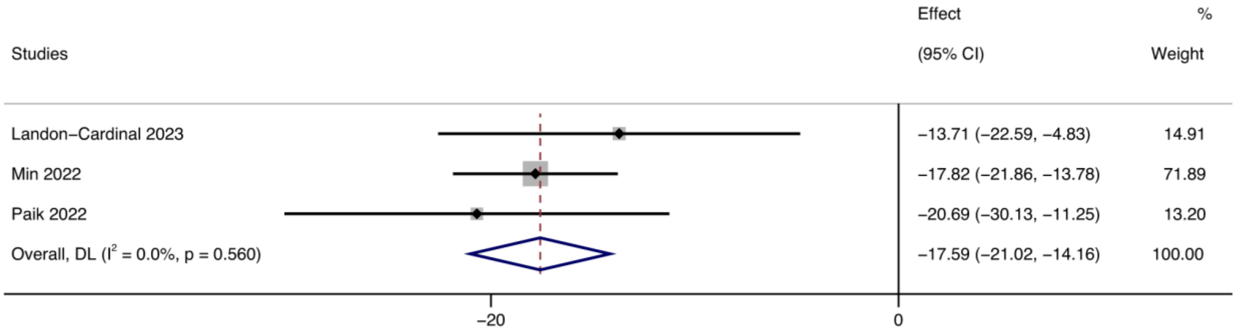

Supplementary Figure 5. Forest plot about the pooled results of CDASI (Without Paik 2021)

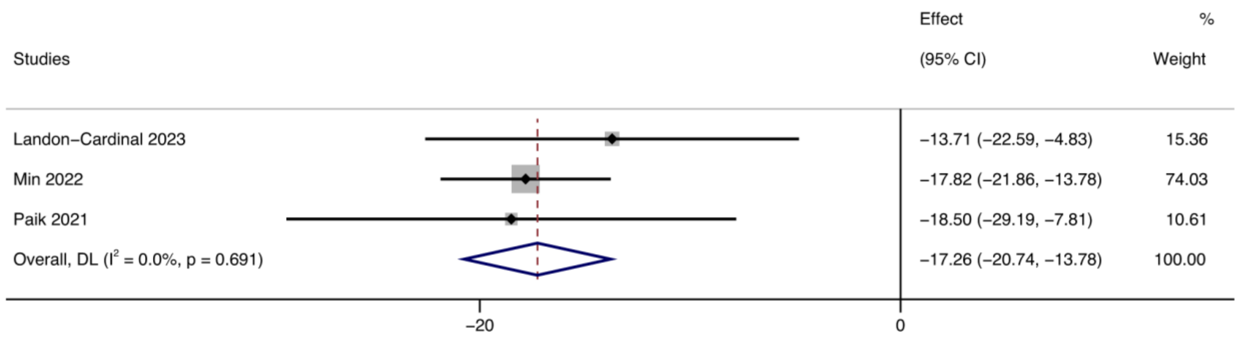

Supplementary Figure 6. Forest plot about the pooled results of CDASI (Without Paik 2022)
